# Informal caregivers during the COVID-19 pandemic perceive additional burden: findings from an ad-hoc survey in Germany

**DOI:** 10.1186/s12913-021-06359-7

**Published:** 2021-04-16

**Authors:** Andrea Budnick, Christian Hering, Simon Eggert, Christian Teubner, Ralf Suhr, Adelheid Kuhlmey, Paul Gellert

**Affiliations:** 1 Charité – Universitätsmedizin Berlin, corporate member of Freie Universität Berlin, Humboldt-Universität Berlin, and Institute of Medical Sociology and Rehabilitation Sciences, Virchowweg 22, 10117 Berlin, Germany; 2 Center for Quality of Care (ZQP), Reinhardstraße 45, 10117 Berlin, Germany

**Keywords:** Informal caregiver, Family caregiver, SARS-CoV-2, COVID-19, Psychosocial burdens

## Abstract

**Background:**

While the relation between care involvement of informal caregivers and caregiver burden is well-known, the additional psychosocial burden related to care involvement during the COVID-19 pandemic has not yet been investigated.

**Methods:**

A total of 1000 informal caregivers, recruited offline, participated in a cross-sectional online survey from April 21 to May 2, 2020. Questionnaires were used to assess COVID-19-specific changes in the care situation, negative feelings in the care situation, problems with implementation of COVID-19 measures, concerns/excessive demands, loss of support, change in informal caregivers’ own involvement in care and problems with provision, comprehension & practicability of COVID-19 information, and to relate these issues to five indicators of care involvement (i.e., being the main caregiver, high expenditure of time, high level of care, dementia, no professional help). Binomial and multiple regression analyses were applied.

**Results:**

Across indicators of care involvement, 25.5–39.7% reported that the care situation rather or greatly worsened during the COVID-19 pandemic, especially for those caring for someone with dementia or those usually relying on professional help. In a multiple regression model, the mean number of involvement indicators met was associated with age (β = .18; CI .10–.25), excessive demands (β = .10, CI .00–.19), problems with implementation of COVID-19 measures (β = .11, CI .04–.19), an increase in caregiving by the informal caregivers themselves (β = .14, CI .03–.24) as well as with no change in the amount of caregiving (β = .18, CI .07–.29) and loss of support (β = −.08, CI −.16–.00). No significant associations with the mean number of involvement indicators met were found for gender, educational level, change in the care situation, negative feelings, and provision, comprehension & practicability of COVID-19 information.

**Conclusion:**

Those caregivers who perceived extensive care burden were those who suffered most during the pandemic, calling for structural support by the healthcare system now and in the future.

**Trial registration:**

This article does not report the results of a health care intervention on human participants.

## Background

Severe acute respiratory syndrome coronavirus 2 (SARS-CoV-2) causing the 2019 novel coronavirus disease (COVID-19) pandemic and the mechanisms determining the cause of COVID-19 are under medical research worldwide [1]; equally, the psychosocial consequences of the restrictions are also the subject of research. To date, studies have mostly addressed adults and older adults [2–4] but informal caregivers have rarely been subject to current pandemic research. An informal caregiver is a person who has a significant personal relationship with, and provides a broad range of assistance for, a person in need of care. It is mostly non-professional and unpaid care [5]. Currently, on average across 18 OECD countries, around 13% of people aged 50 and over report providing informal care at least weekly [6]. In Germany, 4.1 million people are care-dependent, of whom 80% are cared for at home (3.31 million). Two thirds of those cared for at home receive care from an informal caregiver exclusively (2.12 million), and one third together with or exclusively by professional care providers (983,000) [7]. Most people in need of care want to grow old in their home and to be cared for there [8]. The number of people aged between 40 to 85 providing informal care is estimated between 3.0 to 5.2 million people [9]. Although most informal caregivers are motivated by an emotional relationship [10], the task might be linked to physical and psychosocial strain, which may lead to depressive symptoms or reduced quality of life but might also link to potential positive health effects [11–13]. Although there is a small body of literature on the detrimental effects of the pandemic for informal caregivers in long-term care, for instance in the U.S. [14–16], in Italy [17], in the Netherlands [18], and in the UK [19] the literature is not yet comprehensive. Park [14], for example, investigated the fact that long-term caregivers suffer from more psychological distress and somatic symptoms like headaches or fatigue than non-caregivers or short-term caregivers (i.e., providing care for 1 year or less) during the pandemic. Additionally, informal caregivers of persons with dementia who are more concerned about the pandemic were more likely to experience an overload or distress regarding to their role as caregiver [15, 19]. A multicenter online survey regarding psychosocial consequences due to the COVID-19 restrictions (ECLB-COVID19) [20] also confirmed earlier findings from before the pandemic, that informal caregivers have a higher burden regarding mental and physical health. The results also point out that their wellbeing and depression significantly worsened during lockdown compared to non-caregivers [18]. While the numbers of COVID-19 infections during the first wave in spring 2020 in Germany was modest, substantial COVID-19 measures were applied. Throughout those measures, i.e., contact restrictions, informal caregiving still took place [21]. Despite some evidence that psychosocial burden significantly increased for informal caregivers during the pandemic, there is limited evidence on how much that psychosocial burden depends on the involvement and support the informal caregivers received during lockdown. Furthermore, there are no findings on how certain COVID-19-related stressors (i.e., implementation of COVID-19-measures) affect the psychosocial burden of informal caregivers. According to the model of caregiving and the stress process [22], the COVID-19 pandemic and related measures can be seen as an additional contextual stressor in the stress process. Further, this contextual stressor directly relates to primary and secondary stressors such as care involvement, overload, and burnout, which, in turn, are related to secondary stressors, e.g., role strains are viewed as an outgrowth of the ongoing care situation, additional concerns/demands and mediating conditions such as coping or social support, which lead effectively to negative feelings in general felt by caregivers and poor mental health and illness outcomes [22]. However, research is needed to investigate the stress process evaluations of the care situation during the COVID-19 pandemic, assuming more negative evaluations in those caregivers who already had a high care involvement.

### Aims and hypotheses

The aim of the present study was to investigate the relation of COVID-19-related burden and support factors during the coronavirus pandemic with the care involvement of informal caregivers. According to the model of caregiving and stress process [22], we assume that the multiple burden of the pandemic is stronger in those who report higher care involvement. Applying multiple indicators of care involvement (i.e., being the main caregiver, high expenditure of time, high level of care, dementia, no professional help), we hypothesize that care involvement is positively associated with 1) a worsening in the care situation during the pandemic, 2) more concerns/excessive demands, 3) problems with the implementation of COVID-19 measures, 4) loss of support 5) problems with provision, comprehension and practicability of COVID-19 information, 6) more negative feelings, and 7) an increase in caregiving by the informal caregivers themselves, even after controlling for age, gender, and education level.

## Methods

### Study design

We performed an ad-hoc survey with cross-sectional secondary data analysis (data that was collected by someone else for another primary purpose [23]) based on panel data. The panel belongs to the social research and market research institute Forsa (https://www.forsa.de/1/). The study sample was recruited offline and the questionnaire was provided online. Informed consent was obtained from all study participants and people were able to participate from April 21 to May 2, 2020. Contact precautions due to SARS-CoV-2 still applied during that period. For this ad-hoc survey, we followed the Declaration of Helsinki. According to statutes of the ethics committee of the Medical Faculty Charité – Universitätsmedizin Berlin, secondary data analyses do not require ethical approval according to national guidelines (Amtsblatt 230/2019, §2 Abs. 1).

### Study population

The sample consisting of 1000 informal caregivers was recruited offline from a panel of about 80,000 German-speaking people ranging in age from 40 to 85 years. The sample was weighted based on the German Ageing Survey 2014, a representative survey of 40 to 85-year-olds living in private households in Germany [23, 24]. Those who reported taking care of someone aged over 60 for at least 6 months (i.e., inclusion criterion 1) were invited to participate in our survey. Further, the care receiver had to be allocated a level of care according to the compulsory long-term care insurance in Germany (i.e., inclusion criterion 2, from 1 = low level of care to 5 = high level of care [25] and be living at home/ in a community dwelling with or without the informal caregiver (i.e., inclusion criterion 3).

### Measurements

The scales used are based on or informed by established scales and were adapted to the COVID-19 situation by an interdisciplinary team consisting of psychologists, sociologists, a political scientist, a medical doctor, and a health economist who are professionals regarding burdens and challenges of informal caregivers. This is common practice in ad-hoc survey research [26].

#### Background and context of the stress process

The current context is the COVID-19 pandemic in spring 2020. Gender, age, and education level are background or contextual variables according to Pearlin and colleagues [22] and will be considered in our multivariate analyses. Further variables like household size, living conditions of care recipient, occupation, income per month (€), and population size of the place of residence are only used to characterize the study sample.

#### Primary stressor

Five involvement indicators were: main caregiver (yes [indicator met] or no by self-definition), expenditure of time (high [indicator met] = daily for 3 to 6 h or longer or low = once to multiple times a week for a minimum of an hour), level of care (serious level of care [indicator met] = level 3, 4, and 5 or minor level of care = level 1 and 2), dementia in care recipient (yes [indicator met]/no), professional help (day care facility and / or ambulatory care = yes [indicator met]/no). Subsequently, involvement indicators were added up to determine the mean number. The number of involvement indicators ranges from 0 to 5 with higher results indicating a higher involvement in care. In order to determine the level of care (i.e., the third care involvement indicator) on the basis of the German long-term care insurance guidelines according to the German Social Security Code XI, items in six modules contribute to the final score: 1) mobility = 10%; 2) either cognitive and communicative abilities; 3) behavior and psychiatric problem = 15%; 4) self-care = 40%; 5) dealing with requirements because of illness or therapy = 20%; and 6) organization of everyday life and social contacts = 15%), which are weighted differently. The score ranges from 0 to 100, with higher scores indicating worse condition. An applicant receives level of care 1 if the total score is > 12.5, indicating minor impairments of autonomy or of skills; level 2 means considerable impairments, level 3 means serious, level 4 means severe, and level 5 means most severe impairments of autonomy or of skills with 90 to 100 overall points [25].

#### Secondary stressors

##### Change in care situation

To evaluate the change in the care situation during the coronavirus pandemic we asked informal caregivers how their personal care situation developed overall due to the coronavirus situation in the last 4 to 8 weeks on a 3-point Likert scale (much/somewhat better, no change, somewhat/much worse).

##### Concerns/excessive demands

Informal caregivers were asked to state to what extent the following statements apply to them and their care situation on a 4-point Likert scale with response categories ‘applies completely, applies somewhat, applies slightly, does not apply at all’. Items were 1) I’m worried about not being able to cope with domestic nursing care in future; 2) My financial situation has deteriorated due to the combination of care work and the coronavirus situation; 3) I have more work because local services and support structures have been cancelled; 4) The current care situation is excessively stressful for me and 5) The current caregiving situation is excessively stressful for the person I care for. The score ranges from 5 to 20 with higher results indicating more concerns/excessive demands due to COVID-19.

#### Intermediating conditions

##### Problems with implementation of COVID-19 measures

Informal caregivers were asked whether they are able to implement the following recommended COVID-19 measures into domestic nursing care; two answer categories were possible: (1) very well/fairly well and (2) not very well/not at all well. 1) Informing yourself about the latest official hints and recommendations on how to behave in the coronavirus situation; 2) Avoiding direct contact to other people outside your own household as far as possible; 3) Avoiding physical contact with the care receiver as far as possible (e.g., hugging, kissing or touching hands); 4) Washing your hands thoroughly with soap before and after direct contact with the care receiver; 5) Not touching your own face; 6) Explaining the coronavirus situation to the care receiver and/or reassuring them; 7) Wearing a face mask when close in contact to the care receiver, e.g. for personal hygiene; 8) Shifting contact to the care receiver increasingly to phone calls or video calls. The score ranges from 8 to 16 with higher results indicating more problems with implementation of COVID-19 measures.

##### Loss of support

Informal caregivers were asked to state whether in each of the following cases a coronavirus-related change occurred for their care recipient on a 3-point scale: no change (0), decreased (1), has stopped completely (2). The items covered support by 1) other family members or friends; 2) neighbors; 3) ambulatory care service; 4) day care; 5) a 24 h-care worker from Germany; 6) a 24 h-care worker from abroad; 7) other health services (e.g., chiropody); 8) family physician, and 9) other helpers [8, 27]. The score ranges from 0 to 18 with higher results indicating more loss of support due to COVID-19.

##### Provision, comprehension & practicability of COVID-19 information

Informal caregivers were asked whether they agree with the following statements (strongly agree/somewhat agree/somewhat disagree/strongly disagree). The items were 1) I feel that I have access to good public information about coronavirus in Germany; 2) I feel that I can understand the public information about coronavirus and 3) I feel that I can implement the public recommendations on coronavirus effectively in the care situation. The score ranges from 3 to 6 with higher results indicating more problems with provision, comprehension & practicability of COVID-19 information.

#### Stress outcomes

##### Negative feelings

Informal caregivers were asked to state how the current situation affects their feelings (i.e., helplessness, emotionally stressful conflicts with the care receiver, feelings of despair, feelings of anger and rage) in relation to the care situation (i.e., decreased/no change/increased/not specified). Items were adapted from the Sense of Competence Questionnaire (SCQ) [28] which measures different burdens for informal caregivers, and the Geriatric Depression Scale (GDS) [29]. The score ranges from 4 to 12 with higher results indicating more negative feelings due to COVID-19.

##### Caregiving by myself

Informal caregivers where asked whether caregiving by themselves 1) has stopped completely; 2) decreased; 3) hardly changed or 4) increased due to the COVID-19 pandemic.

### Statistical analysis

Characteristics of informal caregivers were presented as mean and standard deviation (SD) for the continuous variable ‘number of involvement indicators met’ and as percentage for categorical variables. We conducted chi-square tests of homogeneity for categorical variables and Welch and t-tests for scales to evaluate differences in the COVID-19 specific constructs between the informal caregivers who met involvement indicators and those who did not. Furthermore, we used binomial logistic regression and multiple regression analysis to assess associations between the COVID-19 specific constructs and each of the dichotomous involvement indicators and the mean number of involvement indicators met. Gender, age and educational level were used as sociodemographic covariates. Nagelkerke R^2^ was used to assess the level of explained variance for the logistic regression models and adjusted R^2^ for the multiple regression model. Analyses were performed using IBM SPSS Statistics for Windows, version 25.0 (IBM Corp., Armonk, NY, USA). *P* values <.05 were considered to indicate statistical significance.

## Results

### Informal caregiver characteristics

In total, *N* = 1000 informal caregivers participated in the survey with a mean (±SD) age of 60 years (±9.3). The proportion of female informal caregivers was 54.7% in the total sample and the majority of informal caregivers had a medium (48.6%) or high-level (35,5%) education (background and context of the stress process). Further variables characterizing the study population are household size (including caregiver), where 51.7% of the informal caregivers stated that they live with another person. 46.9% of the care recipients in this sample live alone. Moreover, 56.1% of informal caregivers were employed and most of them earned between 2500 and 4000 € per month. 55.1% of the total study population lived in a town with a population of 5000 to less than 100,000 people. The highest mean number of involvement indicators was met by caregivers aged between 70 and 79 (*M* = 2.6). Characteristics of informal caregivers according to involvement indicators are shown in Table 1.

**Table 1:** Characteristics of informal caregivers by five involvement indicators (*N* = 1000)

Background & Context	Total % (n)	Main Caregiver		Time required		Level of care		Dementia		Professional help		Number of involvement indicators met
		yes 63.2% (*n* = 632)	no 35.8% (*n* = 358)	Short expenditure of time 84% (*n* = 840)	High expenditure of time 16% (*n* = 160)	Minor Level 47.7% (*n* = 477)	Serious Level 50.8% (*n* = 508)	yes 31.5% (*n* = 315)	no 67.3% (*n* = 673)	yes 52.6% (*n* = 526)	no 47.4% (*n* = 474)	Mean (SD)
*Gender*												
Female	**54.7** (547)	**66.1** (357)	**51.1** (183)	**54.5** (458)	**55.6** (89)	**57.0** (272)	**52.6** (267)	**51.1** (161)	**56** (377)	**52.9** (278)	**56.8** (269)	**2.1** (1.1)
Male	**45.3** (453)	**33.9** (275)	**48.9** (175)	**45.5** (382)	**44.4** (71)	**43.0** (205)	**47.4** (241)	**48.9** (154)	**44** (296)	**47.1** (248)	**43.2** (205)	**2.1** (1.1)
*Age (years)*												
40–49	**12.0** (120)	**10.9** (69)	**14.0** (50)	**13.1** (110)	**6.3** (10)	**10.9** (52)	**13.0** (66)	**10.8** (34)	**12.6** (85)	**11.8** (62)	**12.2** (58)	**2.0** (1.0)
50–59	**38.0** (380)	**35.1** (222)	**43.3** (155)	**41.2** (346)	**21.3** (34)	**40.5** (193)	**36.2** (184)	**42.5** (134)	**36.3** (244)	**39.7** (209)	**36.1** (171)	**2.0** (1.0)
60–69	**31.9** (319)	**31.8** (201)	**31.8** (114)	**31.8** (267)	**32.5** (53)	**31.2** (149)	**31.9** (162)	**27.6** (87)	**33.6** (226)	**33.5** (176)	**30.2** (143)	**2.0** (1.2)
70–79	**16.0** (160)	**19.3** (122)	**10.1** (36)	**11.9** (100)	**37.5** (60)	**15.1** (72)	**16.9** (86)	**17.1** (54)	**15.5** (104)	**13.5** (71)	**18.8** (89)	**2.6** (1.3)
> 80	**2.1 (**21)	**2.8 (**18)	**0.8** (3)	**2.0 (**17)	**2.5 (**4)	**2.3 (**11)	**2.0 (**10)	**1.9 (**6)	**2.1 (**14)	**1.5 (**8)	**2.7 (**13)	**2.4** (1.1)
Mean (SD)	**60.0** (9.3)	**61.1** (9.6)	**58.2** (8.5)	**59.1** (9.0)	**64.8** (9.7)	**60.0** (9.2)	**60.1** (9.5)	**59.9** (9.3)	**60.0** (9.3)	**59.6** (9.0)	**60.5** (9.7)	**2.1** (1.1)
*Education Level*												
low	**13.7** (137)	**14.1** (89)	**12.6** (45)	**11.9** (99)	**23.8** (38)	**12.4** (59)	**14.4** (73)	**14.0** (44)	**13.4** (90)	**13.3** (70)	**14.1** (67)	**2.3** (1.2)
medium	**48.6** (486)	**47.5** (300)	**51.1** (183)	**50.5** (424)	**38.8** (62)	**52.2** (249)	**45.5** (231)	**48.9** (154)	**48.4** (326)	**47.3** (249)	**50.0** (237)	**2.0** (1.1)
high	**35.5** (355)	**36.6** (231)	**33.5** (120)	**35.5** (298)	**35.6** (57)	**33.3** (159)	**38.0** (193)	**35.2** (111)	**35.8** (241)	**36.5** (192)	**34.4** (163	**2.1** (1.1)
not specified	**2.2** (22)	**1.9** (12)	**2.8** (10)	**2.3** (19)	**1.9** (3)	**2.1** (10)	**2.2** (11)	**1.9** (6)	**2.4** (16)	**2.9** (15)	**1.5** (7)	
*Household size (including caregiver)*												
1 person	**17.8** (178)	**16.1** (102)	**20.1** (72)	**19.8** (166)	**7.5** (12)	**20.1** (96)	**15.4** (78)	**14.0** (44)	**19.5** (131)	**20.0** (105)	**15.4** (73)	**1.7** (0.9)
2 persons	**51.7** (517)	**54.6** (345)	**46.6** (167)	**49.2** (413)	**65.0** (104)	**53.5** (255)	**49.4** (251)	**46.0** (145)	**54.2** (365)	**49.8** (262)	**53.8** (255)	**2.1** (1.2)
3 persons	**18.0** (118)	**17.7** (112)	**18.7** (67)	**17.6** (148)	**20.0** (32)	**15.5** (74)	**20.9** (106)	**23.8** (75)	**15.5** (104)	**16.2** (85)	**20.0** (95)	**2.3** (1.1)
> 4 persons	**11.6** (116)	**10.6** (67)	**13.7** (49)	**12.5** (105)	**6.9** (11)	**9.4** (45)	**14.0** (71)	**16.2** (51)	**9.5** (64)	**13.3** (70)	**9.7** (46)	**2.1** (1.1)
not specified	**0.9** (9)	**0.9** (6)	**0.8** (3)	**1.0** (8)	**0.6** (1)	**1.5** (7)	**0.4** (2)	**0.0** (0)	**1.3** (9)	**0.8** (4)	**1.1** (5)	
*Living conditions of care recipient*												
alone	**46.9** (469)	**49.5** (313)	**41.3** (148)	**52.9** (444)	**15.6** (25)	**60.2** (287)	**33.5** (170)	**33.7** (106)	**52.9** (356)	**53.6** (282)	**39.5** (187)	**1.7** (1.0)
with caregiver	**29.6** (296)	**40.2** (254)	**11.5** (41)	**20.2** (170)	**78.8** (126)	**24.1** (115)	**35.4** (180)	**37.8** (119)	**26.0** (175)	**21.3** (112)	**38.8** (184)	**2.9** (1.1)
with another person as caregiver	**23.5** (235)	**10.3** (65)	**47.2** (169)	**26.9** (226)	**5.6** (9)	**15.7** (75)	**31.1** (158)	**28.6** (90)	**21.1** (142)	**25.1** (132)	**21.7** (103)	**1.8** (0.8)
*Occupation*												
employed	**56.1** (561)	**50.8** (321)	**65.6** (235)	**60.8** (511)	**31.3** (50)	**57.4** (274)	**54.7** (278)	**59.4** (187)	**55.0** (370)	**59.3** (312)	**52.5** (249)	**1.9** (1.1)
< 30 h/week	29.2 (164)	32.4 (104)	24.7 (58)	28.0 (143)	42.0 (21)	27.0 (74)	32.0 (89)	35.3 (66)	25.9 (96)	25.3 (79)	34.1 (85)	1.8 (1.2)
> 30 h/week	70.4 (395)	67.0 (215)	75.3 (177)	71.6 (366)	58.0 (29)	72.6 (199)	67.7 (188)	64.2 (120)	73.8 (273)	74.4 (232)	65.5 (163)	2.2 (1.0)
not specified	0.4 (2)	0.6 (2)	0.0 (0)	0.4 (2)	0.0 (0)	0.4 (1)	0.4 (1)	0.5 (1)	0.3 (1)	0.3 (1)	0.4 (1)	2.5 (0.7)
unemployed	**43.3** (433)	**49.2** (311)	**34.4** (123)	**39.2** (329)	**68.8** (110)	**42.6** (203)	**45.3** (230)	**40.6** (128)	**45.0** (303)	**40.7** (214)	**47.5** (225)	**2.3** (1.2)
*Income per month (€)*												
< 1000	**1.9** (19)	**1.9** (12)	**1.4** (5)	**2** (17)	**1.3** (2)	**2.9** (14)	**0.8** (4)	**2.2** (7)	**1.8** (12)	**1.5** (8)	**2.3** (11)	**1.9** (0.9)
1000 – 2500	**28.0** (280)	**28.6** (181)	**26.3** (94)	**27.7** (233)	**29.4** (47)	**27.7** (132)	**27.6** (140)	**26.0** (82)	**28.7** (193)	**28.7** (151)	**27.2** (129)	**2.1** (1.1)
2500 – 4000	**37.1** (371)	**37.5** (237)	**37.4** (134)	**36.2** (304)	**41.9** (67)	**36.9** (176)	**37.8** (192)	**38.1** (120)	**36.8** (248)	**35.7** (188)	**38.6** (183)	**2.2** (1.2)
> 4000	**22.9** (229)	**20.9** (132)	**26.3** (94)	**24.3** (204)	**15.6** (25)	**20.3** (97)	**25.4** (129)	**25.4** (80)	**22.0** (148)	**25.3** (133)	**20.3** (96)	**2.0** (1.1)
not specified	**10.1** (101)	**11.1** (70)	**8.7** (31)	**9.8** (82)	**11.9** (19)	**12.2** (58)	**8.5** (43)	**8.3** (26)	**10.7** (72)	**8.7** (46)	**11.6** (55)	
*Population of home town*												
< 5000	**16.5** (165)	**16.0** (101)	**17.6** (63)	**15.4** (129)	**22.5** (36)	**14.3** (68)	**18.7** (95)	**16.8** (53)	**16.3** (110)	**16.0** (84)	**17.1** (81)	**2.2** (1.2)
5000 - < 20,000	**27.9** (279)	**28.0** (177)	**27.4** (98)	**27.3** (229)	**31.3** (50)	**28.3** (135)	**27.6** (140)	**29.2** (92)	**27.2** (183)	**28.7** (151)	**27.0** (128)	**2.1** (1.2)
20,000 - < 100,000	**27.2** (272)	**26.1** (165)	**29.3** (105)	**27.6** (232)	**25.0** (40)	**26.2** (125)	**28.0** (142)	**26.3** (83)	**27.9** (188)	**28.3** (149)	**25.9** (123)	**2.0** (1.1)
100,000 - < 500,000	**14.7** (147)	**14.9** (94)	**14.5** (52)	**14.9** (125)	**13.8** (22)	**14.0** (67)	**15.4** (78)	**15.9** (50)	**14.1** (95)	**12.5** (66)	**17.1** (81)	**2.2** (1.2)
> 500,000	**13.7** (137)	**15.0** (95)	**11.2** (40)	**14.9** (125)	**7.5** (12)	**17.2** (82)	**10.4** (53)	**11.7** (37)	**14.4** (97)	**14.4** (76	**12.9** (61)	**1.9** (1.1)

*Note.* SD = standard deviation, % = percent, n = number

### Psychosocial burdens for informal caregivers during COVID-19

We further investigated COVID-19-specific constructs across five care involvement indicators (see Table 2). Significant results are presented below. Across indicators, caregivers mostly reported no change in the care situation. However, a substantial proportion of those who cared for a person with dementia (39.7%) and those that usually relied on professional help (34.8%) reported rather or greatly worsening burdens during the COVID-19 pandemic compared to their counterparts. Looking at other aspects, more caregivers not taking care of someone with dementia (69.4%) or not relying on professional help (70.3%) reported that there was no change in care situation compared to their counterparts. Informal caregivers taking care of someone with dementia and usually relying on professional help showed more negative feelings, concerns/excessive demands, and loss of support, in contrast to their counterparts. Caregivers of a person with dementia reported more problems with implementation of COVID-19 measures with a mean of 10.1 (±2.0), and with provision, comprehension, and practicability of COVID-19 information of 3.4 (±0.8). More informal caregivers without professional help stated that there was no change in how much care they gave their care receiver during the COVID-19 pandemic (71.3%) in contrast to those with professional help (63.3%). Those who are the main caregiver and invest a large amount of time stated more often that they gave their care receiver even more care during the COVID-19 pandemic (21.7%; 21.3%) compared to those not meeting those involvement indicators. None of the informal caregivers stated that caregiving stopped completely due to pandemic. Finally, informal caregivers taking care of someone with a serious level of care showed more concerns/excessive demands with a mean of 10.2 (±3.3), problems with implementation of COVID-19 measures with 9.9 (±2.0), and loss of support with 2.5 (±2.6) in contrast to those with a minor care level.

**Table 2:** Burdens for informal caregivers during the first wave of the COVID-19 pandemic (*N* = 1000)

	Main Caregiver		Time required		Level of care		Dementia		Professional help		Number of Involvement indicators met
	Yes 63.2% (*n* = 632)	No 35.8% (*n* = 358)	Short expenditure of time 84.0% (*n* = 840)	High expenditure of time 16.0% (*n* = 160)	Minor Level 47.7% (*n* = 477)	Serious Level 50.8% (*n* = 508)	Yes 31.5% (*n* = 315)	No 67.3% (*n* = 673)	Yes 52.6% (*n* = 526)	No 47.4% (*n* = 474)	Mean (SD)
***Secondary Stressors***											
*Change in care situation (%, n)*											
much/somewhat better	3.3 (21)	3.1 (11)	3.1 (26)	3.8 (6)	3.1 (15)	3.1 (16)	2.2 (7)	**3.7** (25)***	**3.6** (19)**	1.5 (7)	2.0 (1.1)
no change	63.9 (404)	65.4 (234)	64.8 (544)	63.1 (101)	68.3 (326)	61.2 (311)	56.2 (177)	**69.4** (460)***	59.3 (312)	**70.3** (333)**	2.1 (1.1)
somewhat/much worse	31.3 (198)	29.1 (104)	30.4 (255)	30.6 (49)	27.3 (130)	33.5 (170)	**39.7** (125)***	26.2 (176)	**34.8** (183)**	25.5 (121)	2.2 (1.1)
n.s.	1.4 (9)	2.6 (9)	1.8 (15)	2.5 (4)	1.3 (6)	2.2 (11)	**1.9** (6)***	1.8 (12)	**2.3** (12)**	1.5 (7)	2.0 (1.1)
*Concerns/excessive demands (M, SD)*	9.9 (3.4)	9.7 (3.3)	9.8 (3.3)	10.2 (3.9)	9.5 (3.4)	**10.2** (3.3)**	**10.7** (3.6)***	9.4 (3.2)	**10.3** (3.5)***	9.3 (3.2)	2.1 (1.1)
***Intermediating Factors***											
*Problems with implementation of COVID-19 measures (M, SD)*	9.8 (1.9)	9.7 (1.8)	9.7 (1.8)	10.1 (2.3)	**9.6 (1.7)**	**9.9** (2.0)**	**10.1** (2.0)***	**9.6 (1.8)**	9.8 (1.9)	9.7 (1.8)	2.1 (1.1)
*Loss of support (M, SD)*	2.2 (2.5)	2.4 (2.4)	2.2 (2.4)	2.1 (2.9)	1.9 (2.3)	**2.5** (2.6)***	**2.7** (2.7)***	2.0 (2.3)	**2.9** (2.7)***	1.5 (2.0)	2.1 (1.1)
*Provision, comprehension & practicability of COVID-19 information*^*5*^ *(M, SD)*	3.3 (0.7)	3.3 (0.8)	3.3 (0.8)	3.3 (0.7)	3.3 (0.7)	3.3 (0.8)	**3.4** (0.8)**	3.3 (0.7)	3.4 (0.8)	3.3 (0.7)	2.1 (1.1)
***Stress Outcomes***											
*Negative feelings M (SD)*	8.7 (1.6)	8.7 (1.7)	8.7 (1.6)	8.8 (1.8)	8.7 (1.6)	8.7 (1.7)	**9.0** (1.8)***	8.6 (1.5)	**8.8** (1.6)**	8.6 (1.6)	2.1 (1.1)
*Caregiving by myself (%, n)*											
has stopped completely	0.0 (0.0)	0.0 (0.0)	0.0 (0.0)	0.0 (0.0)	0.0 (0.0)	0.0 (0.0)	0.0 (0.0)	0.0 (0.0)	0.0 (0.0)	0.0 (0.0)	–
decreased	9.5 (60)	**18.4** (66)***	**14.6** (123)***	2.5 (4)	11.5 (55)	13.8 (70)	12.4 (39)	13.1 (88)	**15.2** (80)*	9.9 (47)	1.7 (0.9)
did not change	**68.8** (435)***	63.7 (228)	65.4 (549)	**76.3** (122)***	66.9 (319)	67.3 (342)	64.4 (203)	68.4 (460)	63.3 (333)	71.3 (338)*	2.1 (1.2)
increased	**21.7** (137)***	17.9 (64)	20.0 (168)	**21.3** (34)***	21.6 (103)	18.9 (96)	23.2 (73)	18.6 (125)	**21.5** (113)*	18.8 (89)	2.1 (1.1)

*Note.* Significant values are shown in bold type. *p < .05, ** *p* < .01 und *** *p* < .001; M = mean, SD = standard deviation, % = percent, n = number, > = greater than, < = less than

### Multivariate results for the association between informal caregivers’ characteristics and psychosocial burdens during COVID-19 and care involvement of informal caregivers

Results indicate that being the main caregiver was significantly associated to the age of the caregivers (OR = 1.04; 95% CI 1.02–1.06). Further, it was significantly associated to an increase in caregiving duty by the informal caregivers themselves (OR = 2.46; 95% CI 1.49–4.06) as well as to no change in how much they care they provided during COVID-19 (OR = 2.22; 95% CI 1.42–3.49). A high expenditure of time spent on informal caregiving was also significantly related to the age of the caregivers (OR = 1.07; 95% CI 1.05–1.09), to an increase in caregiving by the informal caregivers themselves (OR = 5.05; 95% CI 1.70–15.05), and to no change in how much care they provided during COVID-19 (OR = 5.45; 95% CI 1.89–15.73). A high level of care was significantly related to concerns/excessive demands (OR = 1.10; 95% CI 1.01–1.11), problems with implementation of COVID-19 measures (OR = 1.11; 95% CI 1.03–1.20), and loss of support (OR = 1.09; CI 1.02–1.16) during COVID-19. Taking care of someone with dementia was significantly related to concerns/excessive demands (OR = 1.06; 95% CI 1.00–1.12) and problems with implementation of COVID-19 measures (OR = 1.06; 95% CI 1.02–1.20). Lastly, having no professional help for caregiving was significantly related to loss of support (OR = 0.76; 95% CI 0.71–0.82; Table 3). The explained variance was highest for the involvement indicator high expenditure of time (Nagelkerke R^2^ = 14.80%). There were no significant associations between gender, change in care situation, negative feelings or problems with provision, comprehension and practicability of COVID-19 measures with one of the involvement indicators.

**Table 3:** Associations between characteristics of informal caregivers and psychosocial burdens during COVID-19 and involvement of informal caregivers

	Logistic Regression					Multiple Regression
***Primary Stressors***	**Main Caregiver**^**a**^	**High expenditure of time**^**b**^	**High level of care**^**c**^	**Dementia**^**d**^	**No professional help**^**e**^	**Number of involvement indicators met**^**f**^
	OR (95% CI)	OR (95% CI)	OR (95% CI)	OR (95% CI)	OR (95% CI)	β (95% CI)
***Background & Context***						
*Age*	1.04 (1.02–1.06)***	1.07 (1.05–1.09)***	1.00 (0.99–1.02)	1.01 (1.00–1.03)	0.99 (0.98–1.01)	.18 (0.10–0.25)***
*Gender*						
men	0.81 (0.61–1.09)	0.97 (0.67–1.40)	1.20 (0.91–1.58)	1.23 (0.92–1.65)	0.77 (0.58–1.01)	−.01 (−.08–.07)
*Education Level*						
low	0.91 (0.57–1.45)	1.36 (0.80–2.30)	0.90 (0.58–1.40)	0.91 (0.57–1.47)	1.03 (0.66–1.61)	.01 (−.07–.09)
medium	0.86 (0.63–1.17)	0.71 (0.47–1.08)	0.74 (0.55–0.99)*	1.04 (0.76–1.43)	1.06 (0.78–1.43)	−.04 (−.12–.04)
high	1	1	1	1	1	1
***Secondary Stressors***						
*Change in care situation*						
much/somewhat better	1	1	1	1	1	1
no change	1.02 (0.47–2.22)	0.78 (0.27–1.87)	0.86 (0.40–1.83)	1.21 (0.50–2.91)	1.87 (0.87–4.04)	.04 (−.12–.21)
somewhat/much worse	1.36 (0.59–3.11)	0.83 (0.29–2.33)	0.90 (0.40–1.99)	1.63 (0.65–4.10)	1.93 (0.85–4.40)	.11 (−.06–.28)
*Concerns/Excessive demands*	1.04 (0.98–1.09)	1.06 (0.99–1.13)	1.10 (1.01–1.11)*	1.06 (1.00–1.12)*	0.97 (0.92–1.02)	.10 (.00–.19)*
***Intermediating Factors***						
*Problems with implementation of COVID-19 measures*	0.99 (0.92–1.08)	1.10 (0.99–1.22)	1.11 (1.03–1.20)**	1.06 (1.02–1.20)*	1.01 (0.94–1.10)	.11 (.04–.19)**
*Loss of support*	0.97 (0.91–1.03)	0.99 (0.91–1.08)	1.09 (1.02–1.16)*	1.06 (0.99–1.13)	0.76 (0.71–0.82)***	−.08 (−.16–.00)*
*Problems with provision, comprehension & practicability of COVID-19 information*	0.95 (0.78–1.16)	0.93 (0.71–1.21)	0.84 (0.68–1.01)	1.01 (0.83–1.24)	1.02 (0.83–1.24)	−.06 (−.13–.02)
***Stress Outcomes***						
*Negative feelings*	1.00 (0.90–1.11)	1.07 (0.93–1.23)	0.95 (0.86–1.05)	1.06 (0.95–1.18)	0.98 (0.86–1.09)	.02 (−.07–.10)
*Caregiving by myself*						
decreased	1	1	1	1	1	1
almost no change	2.22 (1.42–3.49)***	5.45 (1.89–15.73)**	0.97 (0.62–1.51)	1.25 (0.77–2.02)	0.99 (0.63–1.57)	.18 (.07–.29)**
increased	2.46 (1.49–4.06)***	5.05 (1.70–15.05)**	0.74 (0.45–1.21)	1.13 (0.67–1.91)	1.10 (0.66–1.84)	.14 (.03.-.24)*

*Note. OR* Odds ratio, *CI* Confidence interval. β = standardized coefficient. Significant values are shown in bold type. *p < .05, ** *p* < .01 and *** *p* < .001, ^a^ Nagelkerke R^2^ = 7.20%, ^b^ Nagelkerke R^2^ = 14.80%, ^c^ Nagelkerke R^2 =^ 5.50%, ^d^ Nagelkerke R^2^ = 7.10%, ^e^ Nagelkerke R^2^ = 13.30%, ^f^ R^2^ = 0.07, Adjusted R^2^ = 0.05

Finally, we conducted a multiple regression analysis with the number of involvement indicators met, regressed on the caregiver’s characteristics and the COVIC-19-specific assessments. We found age (β = .18; 95% CI .10–.25), concerns/excessive demands (β = .10; 95% CI .00–.19), problems with implementation of COVID-19 measures (β = .11; 95% CI .04–.19), an increase in caregiving by the informal caregivers themselves (β = .14; 95% CI .03–.24), no change in how much care they provided (β = .18; 95% CI .07–.29), and loss of support (β = −.08; 95% CI −.16–.00) during COVID-19 to be significantly related to the mean number of involvement indicators met (Table 3, last column), *F* (13, 927) = 4.96, *p* < .001.

## Discussion

In the present ad-hoc survey, we hypothesized that higher levels of care involvement during the COVID-19 pandemic are associated with COVID-19-related indicators of care stress: 1) a worsening in the care situation during the pandemic, 2) more negative feelings, 3) more concerns/excessive demands, 4) problems with the implementation of COVID-19 measures 5) problems with provision, comprehension and practicability of COVID-19 information 6) an increase in caregiving by the informal caregivers themselves and 7) loss of support even after controlling for age, gender, and education level. As outlined by Pearlin and colleagues [22] the caregiving and stress process in the lives of caregivers moves forward over time. They recommend using their model as a heuristic device rather than a literal reflection of realities. Thus, this model is an appropriate framework to better understand the web of conditions linked under the current COVID-19 pandemic that vary in their impact on caregivers’ health and behavior.

More than half of the present study population of caregivers are women with an average age of about sixty years and medium education level, which is much in accordance with the official data from the German national nursing care statistics [7]. The current findings indicate that almost one third (31.1%) of the main caregivers reported a worsening in the care situation due to the COVID-19 pandemic; however, the situation rather or greatly worsened for those who care for a person with dementia (39.7%) and among those who receive professional help (34.8%). At the same time, informal caregivers of a person with dementia and those receiving professional help perceived a higher burden compared to their counterparts in different aspects like negative feelings, concerns/excessive demands, and loss of support. Caregivers of a person with dementia also had more problems with the implementation of COVID-19 measures and the provision, comprehension, and practicability of COVID-19 information. As hypothesized, especially informal caregivers of a person with dementia seem to be a group perceiving an additional burden during the COVID-19 pandemic. This finding is in line with previous studies as cognitive impairment, independent of a particular situation such as a pandemic, might increase subjective burden among informal caregivers [12, 30]. Moreover, any change in daily routines, e.g. implementation of hygiene measures, change in support or other changes, might cause stress for people with dementia and even the caregivers [19, 31, 32]. Therefore, our findings suggest that existing support structures urgently need to be maintained and the need for support might be even higher during the COVID-19 pandemic than before among people with dementia and their caregivers. Specific COVID-19 care service offers would be able to buffer parts of the additional burden.

Our analyses show that main caregivers were more than twice as likely to either increase caregiving activities or not change anything during the COVID-19 pandemic in spring 2020. An increase in caregiving by the informal caregiver might be caused by loss of support services or retreat of the individual network. This is even clearer in the subgroup of those already spending a large amount of time (i.e., at least 3 to 6 hours daily) on caring activities; their effort either increased with about five-fold likelihood or did not change. Thus, it should not be taken for granted that main caregivers or those already spending a large amount of time on caregiving activities take over even more caregiving tasks. The risk for burn-out or depression might increase immediately, and support structures should especially focus on the mental wellbeing of caregivers, rather than providing financial help.

Findings further indicate that with every year of age, a caregiver has a slightly increased risk of spending more time on caregiving. However, our data do not tell us if this is caused by the caregivers’ aging progress or the care receivers’ worsening health condition or both. Moreover, results underline informal caregivers’ additional burden during the pandemic in spring 2020 for those caregivers caring for someone with a high level of care or with dementia. They have a slightly increased risk for concerns, excessive demands, and problems with implementation of COVID-19 measures, indicating remarkable challenges and the need for reliable support structures to be provided by the German health care system. Those caregivers without professional help had a lower risk for loss of support in general, likely because they do the main portion of the daily care work already without any help. Even that group of informal caregivers might need professional support during the second wave of the pandemic and beyond. Finally, concerning the number of care involvement indicators, within the multivariate analysis we found the caregiver’s age to be substantially associated with a higher number of care involvement indicators. Further, substantial associations were found for more concerns/excessive demands, problems with the implementation of COVID-19 measures, loss of support, almost no change in ‘caregiving by myself’ and an increase in that as well. Therefore, it is an essential task for health professionals to mitigate these factors during the second wave of the COVID-19 pandemic and beyond through a better approach to available information on the implementation of COVID-19 measures. Moreover, staying in close contact via telephone or video conferences with friends and relatives might be important to discuss concerns and reduce different facets of lost support. A daily structure including caregiving activities and time outs for physical and mental recreation is even more important during the COVID-19 pandemic than before.

### Strengths and limitations

To our knowledge, this was the first ad-hoc survey about the burden faced by and resources of informal caregivers due to the COVID-19 pandemic in spring 2020 in Germany. The ad-hoc survey allowed us to obtain standardized answers from 1000 informal caregivers aged 40 to 85 years immediately during contact restrictions. Additionally, our results are valuable for the current upcoming challenges for informal caregivers during the so-called second wave of the COVID-19 pandemic and beyond. However, the study’s limitations need to be addressed. Although the genuine population was recruited offline, our current survey comprised a weighted ad-hoc online survey that may limit generalizability to the entire population of informal caregivers in Germany. Thus, informal caregivers with limited access to internet or web-enabled devices might not have taken part in this survey. Moreover, the COVID-19 pandemic has shown regional variations, but our data do not differentiate for regions or hotspots. Finally, the unexpected event of the COVID-19 pandemic followed by our ad-hoc survey did not allow us to use validated assessment instruments focusing on the additional burden of caregiving. However, it is common practice in ad-hoc surveys to adapt existing validated instruments [26]. As a limitation, our assessments of psychosocial stresses can only be interpreted as initial indications of caregiver stress during a pandemic. Clinically relevant screening of a diagnosis of dementia or depression was beyond the scope of this proposal, but should be taken into account in future studies of stress and burden of caregivers in situations with additional external macro-level changes such as a pandemic.

## Conclusion and future directions

Our findings shed light on the additional burden experienced by informal caregivers due to the COVID-19 pandemic in spring 2020 in Germany. Overall, the situation deteriorated for both the main informal caregiver and the care receiver. As informal caregivers are a main pillar of many care systems around the world, who have continued to be involved rather than relinquishing responsibilities during the COVID-19 pandemic, the daily service they provide for care recipients and for society is essential and must be supported by an incoming support structure, in order to maintain the caregivers’ health and safeguard ambulatory informal care during future waves of the COVID-19 pandemic or other major crises. Our findings yield the recommendation that all institutions with a mandate to support informal caregivers (e.g., general practitioners or nursing care services) should be supported with protective clothing, regular testing for SARS-CoV-2-RNA, and the time for appropriate hygiene measures to be able to protect others and themselves and to maintain their work throughout the COVID-19 pandemic. Special attention should be paid to informal caregivers of persons with dementia and those without professional help, inter alia to avoid an increase in elder abuse or neglect that might be negative side effects of the COVID-19 pandemic. Thus, the focus should be on supporting the mental wellbeing and social participation of the caregivers along with measures that protect the caregivers and their relatives from COVID-19. In conclusion, support structures for informal caregivers should be kept running during crises now and in the future.

## Data Availability

The dataset used for the current ad-hoc survey is available from the corresponding author on reasonable request.
